# Is the re-use of sterilized implant abutments safe enough? (Implant abutment safety)

**DOI:** 10.4317/medoral.22967

**Published:** 2019-08-19

**Authors:** Mª Angeles Sánchez-Garcés, Marta Jorba, Joan Ciurana, Miguel Vinas, Mª Teresa Vinuesa

**Affiliations:** 1 PhD, MD. Aggregate Professor Department of Dentistry. Faculty of Medicine and Health Sciences IDIBELL. University of Barcelona, Campus Bellvitge, Barcelona. Spain; 2 Ms in Microbiology. Department of Pathology & Experimental Therapeutics, Faculty of Medicine and Health Sciences. University of Barcelona and IDIBELL. Campus Bellvitge, Barcelona. Spain; 3 Ms Student. Department of Dentistry. Faculty of Medicine and Health Sciences IDIBELL. University of Barcelona, Campus Bellvitge, Barcelona. Spain; 4 PhD. Chairman in Microbiology. Department of Pathology & Experimental Therapeutics, Faculty of Medicine and Health Sciences. University of Barcelona and IDIBELL. Campus Bellvitge, Barcelona. Spain; 5 PhD, MD. Associate Professor. Department of Pathology & Experimental Therapeutics, Faculty of Medicine and Health Sciences. University of Barcelona and IDIBELL. Campus Bellvitge, Barcelona. Spain

## Abstract

Background: The reuse of implant healing abutments is common in dental practice. Effective elimination of bacteria and viruses is accomplished by conventional sterilization. The aim of this work was to explore the eventual survival of microorganisms on sterilized healing abutments and to rule out the presence of transmissible organic material after standard procedures. Material and Methods: A total of 55 healing abutments previously used in patients will be washed and sterilized in a steam autoclave at 121ºC for 15 min. Each healing abutment will be cultured in Brain Heart Infusion broth (BHI) under strict aseptic conditions. Besides, two control groups will be included: one of 3 unused healing abutments, and the other of just medium. After 10 days at 37°C under a 5% CO2 100 µl of the broth will be plated on solid media (Brain Infusion Agar, BHIA) and Columbia Blood agar to test for sterility. The remaining volume will be centrifuged, the sediment fixed, and a Gram stain performed to discard the presence of non-cultivable microorganisms. Moreover, to determine the presence of remaining organic material after the cleaning and sterilizing treatments, the bioburden will be determined by measuring total organic carbon (TOC) in another 10 previously used healing abutments, cleaned and sterilized, that will be submerged in Milli-Q water and sonicated. Results: No bacterial growth was detected on any of the 58 cultured abutments, indicating that the sterilization was completely satisfactory in terms of removal of live bacteria or spores. Nevertheless, significant amounts of organic carbon may still be recovered (up to 125,31 µg/abutment) after they have been sterilized. Conclusions: Significant amounts of the bioburden remained adhered to the surfaces in spite of the cleaning and sterilization procedures. Taking into account our results and data from other authors, the presence of infectious particles on the reused healing abutments such as prions cannot be ruled out.

** Key words:**Healing abutment, abutment surface, peri-implantitis, mucositis, sterilization.

## Introduction

The reuse of implant abutments is common practice, as it reduces costs for both patients and dentists. Since abutments are mostly made of titanium, it has been assumed that autoclave sterilization guarantees the safety of such reuse, although it can alter the composition of the surface due to atmospheric pollutants, especially when the sterilization process is repeated several times (1).

Abutments should favor the maturation of peri-implant tissues during osseointegration, favouring the modeling of soft tissues surrounding the implant. Moreover, the attachment of soft tissue around the implant abutments takes place through the establishment of a hemidesmosomal junction involving inflammatory cells (about 3 mm thickness) that contributes to osseointegration.

Prevention of the presence of bacteria in the region together with the use of sterile instruments and components to avoid cross-infection between patients are primary goals of implantology. Thus, despite the common practice of reutilization, most manufacturers recommend just a single use (2). Several authors have concluded that the use of sterilization procedures alone for the treatment of reused abutments might not be sufficient, and recommend previous cleaning protocols to detach incrusted material before a re-sterilization step (3)

Different strategies have been assayed to minimize the consequences of reutilization, including the use of cheaper materials, such as glass-fiber (4,5), and different cleaning processes (6-8).

The aim of this work was to explore the eventual survival of microorganisms on sterilized abutments, as well as to explore to what extent bacteria found on abutments could be the direct cause of implant failures. There is a high proportion of initial implant success, and later failure is normally attributed to biomechanical or microbiological causes (2-9). Thus, our aim can be extended to answer a new question: To what extent does biological detritus on reused abutments contribute to implant failure? 

## Material and Methods

A total of 55 healing abutments previously used in one or more patients during the passive osseointegration period of three to six months were provided by eight different dental clinics of Barcelona, Spain. Once retired, the healing abutments were processed for cleaning and disinfection by immersion in enzymatic detergent for 2-5 minutes, followed by an ultrasound bath at 40-45ºC for 10-15’ and finally dried. They were placed in heat-sealed sterilization bags (3M Steri-Dual ECO, Ref: 8652). The sterilization was carried out with a Class B autoclave in a program for metals (134ºC, 12 minutes, 2.1 bar pressure).

-Sample culture

**Figure 1:**
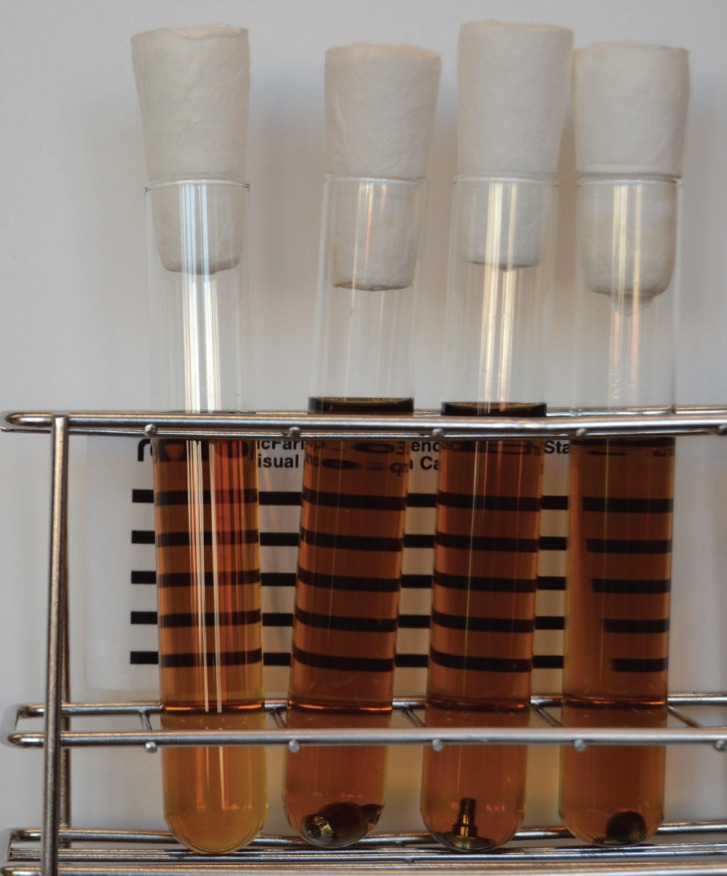
Microbiological cultures. 4 tubes containing 10 ml of Brain Hearth broth, the left one been the negative control with only medium and the other ones with 3 healing abutments, after incubation at 37°C for 10 days under 5% CO2 atmosphere. No differences in turbidity were observed.

Each healing abutment was removed from the sterilization bag and submerged in a rich microbiological medium (10 mL of Brain Heart Infusion broth (BHI) Sharlau, Sentmenat, Spain) in 18x180 mm test tubes under strict aseptic conditions. Two control groups were prepared: the first consisted of three new (unused) healing abutments (Nobel Biocare, Sweeden; Ref: 33445 BmK Syst RP; Sterilized using irradiation); and the second, of just the bacteriological medium (lacking an abutment). The tubes were incubated for 10 days, at 37°C under a 5% CO2 atmosphere and examined visually daily for turbidity. At the end of the experiment, the tubes were examined again and Petri dishes containing BHI agar (BHIA) and Columbia Blood agar (Sharlau, Sentmenat, Spain) were inoculated with 100 µl of the medium from each tube to test for sterility. The contents of each tube were centrifuged at 2.500 rpm and sediment was Gram stained and observed at 1000 x’ to discard the presence of non-cultivable microorganisms (Fig. 1).

-Determination of Total Organic Carbon (TOC)

Moreover, to determine the presence of remaining organic material after the cleaning and sterilizing treatments, the bioburden present in the used abutments was determined by measuring total organic carbon (TOC) present.

A different stock of 10 previously used healing abutments, cleaned and sterilized, were submerged in 10 Eppendorf flasks with 1.5 ml Milli-Q water and sonicated in an ultrasonic bath for 1 hour.

A volume of 15 ml Milli-Q water was used as blank control.

TOC was measured using a multi N/C® 3100 analyzer (Analytic Jena, Jena, Germany).

## Results

-Sample culture

None of the 55 used abutments processed in the microbiological experiments produced obvious visible turbidity in the medium, although in some cases microparticulated material appeared after 24 hours of incubation (first examination) and remained throughout the incubation period. However, it should be stated that in none of these samples did the turbidity increase over this period. Moreover, after 10 days of incubation and the subsequent inoculation of aliquots of the liquid medium onto BHIA and Columbia Blood agar plates, no bacterial growth was detected on any of them, indicating that the sterilization of the used abutments was completely satisfactory in terms of removal of live bacteria or spores. In some cases, the Gram stain examination of the smear was difficult to interpret because of the presence of abundant debris stained with a pink color.

-Determination of Total Organic Carbon (TOC)

The TOC determinations of the abutments were much higher than expected ( Table 1). It is worth noting that a significant amount of organic material was recovered from the used abutments, indicating that it had remained adhered to the surfaces of the abutments. Since there is a huge difference between the organic carbon content of the abutments and the Milli-Q water used for submerging the abutments prior to sonication, our results demonstrate that significant amounts of the bioburden remained adhered to the surfaces.

**Table 1:**
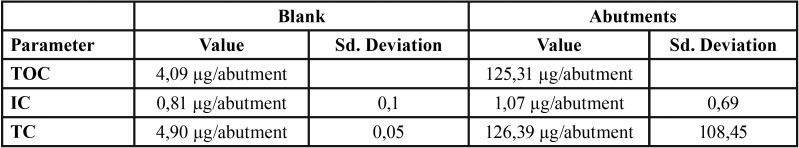
Values of Carbon adhered to used abutments. TOC: Total Organic Carbon, IC: Inorganic Carbon , TC: Total Carbon (TC = IC+TOC).

## Discussion

Although no living cells survive the autoclaving, it is well known that many molecules as well as epithelial and blood cells and bacterial fractions may retain certain properties after the sterilization procedure. The organic material found on the reused abutments would come from the patient in which the abutment was connected to. Subsequently, it may either affect cell adhesion and the effective spreading and attachment of epithelium and connective tissue(2,10), or promote inflammatory processes in a hypothetical re-receptor. The proteins and amino acids that can remain adhered to titanium are extremely difficult to remove.

Sterilization of previously used or intentionally contaminated healing abutments is a safe procedure to eliminate bacteria, virus and fungi as different studies had demonstrated previously (1) .

Given that recent studies suggest that the sterilization of the healing abutments does not eliminate all the biological debris, the placement of contaminated abutments could be associated with the development of peri-implant disease allowing a good place to adhere and grow (11-13).

All this represents different kinds of health risk for the second user. One of these risks may be illustrated by considering prions. Biological debris could act as an effective vector for the transmission of prions. These infectious proteinaceous agents can cause neurodegenerative diseases that in humans include Kuru, Creutzfeldt-Jakob disease, Gerstmann-Straussler-Scheinker disease, and Fatal Familial Insomnia. In principle, the measurement of “viability” when considering prions should be regarded as the measurement of the maintenance of infectivity. Despite only a few laboratories in the world are undertaking experimental work with prions, notably that of Stanley B. Prusiner (Nobel Prize in Physiology or Medicine, 1997), the work has led to several major concerns (14). The first and most relevant in the current context is that prions need to be completely inactivated using harsher conditions than those used against bacteria and viruses. To ensure prion inactivation, the thermal sterilization should be combined with chemical treatment. It would appear that procedures used for routine sterilization of surgical instruments cannot inactivate prions (15,16), which already led to the development of new and more stringent recommendations for reprocessing instruments and these should eventually be applied to abutments (17). This has been reinforced by the discovery that prions that are responsible for bovine spongiform encephalitis (BSE) can be up to 1 million times more difficult to inactivate than the most commonly used hamster prions; thus, one cannot exclude the possibility that human prions are also much more resistant than the laboratory prions (10). These recommendations are based on conventional autoclaving (121ºC) combined with chemical attack; this may be achieved by autoclaving in the presence of 1 M sodium hydroxide, or by soaking in 2% bleach for 1 h. Such treatments are extremely corrosive and may cause irreversible damage to the surface of abutments (18).

Prevalence of asymptomatic Creutzfeldt-Jakob disease (CJD) in UK population in people born from 1941 to 1985 is 1:2000 and prion iatrogenic transmission (blood transfusions, organ transplants and surgical instrumentation) is therefore possibility. Another source of prions could be bovine bone substitutes used widely for bone regeneration after or simultaneously to the dental implant placement. These materials keep some proteins, their manufacturing processes are not guaranty to the inactivation of the prion, and in consequence, Kim *et al.* (19) suggest abolishing the use of bovine bone.

The presence of organic carbon reported in our study means that organic material originating in the patient is adhered to the surface and, subsequently, the presence of prions cannot be ruled out. In conclusion, we believe that, despite costs, the practice of reusing implant abutments should be abandoned, since it cannot be demonstrated to be safe enough.

Further studies trying to identify the source of the organic carbon adhered in the abutments are needed. In addition, it is worth elucidating if there could be any safe procedure to effectively remove all the organic material present in the titanium surfaces of the reused healing abutments.
